# *Ureaplasma Urealyticum* Infection Contributes to the Development of Pelvic Endometriosis Through Toll-Like Receptor 2

**DOI:** 10.3389/fimmu.2019.02373

**Published:** 2019-10-04

**Authors:** Eui Jeong Noh, Dong Jae Kim, Jun Young Lee, Jong Hwan Park, Jong-Seok Kim, Jae Won Han, Byoung Chan Kim, Chul Jung Kim, Sung Ki Lee

**Affiliations:** ^1^Department of Obstetrics and Gynecology, College of Medicine, Konyang University, Daejeon, South Korea; ^2^Laboratory Animal Resource Centre, Daegu Gyeongbuk Institute of Science & Technology (DGIST), Daegu, South Korea; ^3^Laboratory Animal Medicine, College of Veterinary Medicine, Chonnam National University, Gwangju, South Korea; ^4^Myunggok Medical Research Institute, College of Medicine, Konyang University, Daejeon, South Korea; ^5^Korean Collection for Type Cultures, Biological Resource Center, Korea Research Institute of Bioscience and Biotechnology, Daejeon, South Korea

**Keywords:** *Ureaplasma urealyticum*, endometriosis, mesothelial cells, infection, inflammation, Toll-like receptor 2

## Abstract

Endometriosis is a chronic gynecological disorder, characterized by the presence of ectopic endometrial tissue outside the uterine cavity. Among several hypotheses, Sampson's theory of retrograde menstruation is still applicable. Recent studies have reported the importance of inflammation among endometrial tissue, the peritoneum, and immune cells. However, less is known regarding the role of bacterial infection in the pathophysiology of endometriosis. We hypothesized that *Ureaplasma urealyticum* infection might contribute to the development of endometriosis by inducing the production of inflammatory mediators by peritoneal mesothelial cells (PMCs), possibly through TLR2. Hence, our objective was to reveal whether PMC infection by *U. urealyticum* is associated with endometriosis. Moreover, we aimed to demonstrate the molecular mechanism involved in this relationship. We developed a new infection-induced mouse model of endometriosis with wild type and Tlr2-deficient mice. Based on the *in vivo* mouse model, *U. urealyticum*-infected mice showed significantly increased numbers and sizes of ectopic endometriotic lesions. *U. urealyticum* upregulated not only the production of IL-6, CXCL1, and CCL2, but also the expression of ICAM-1, VCAM-1, and MMP2 in murine PMCs. Similarly, endometrial stromal cells dose-dependently produced IL-6, CXCL1, and CCL2 in response to *U. urealyticum* infection. The series of inflammatory responses in PMCs was mediated mainly through TLR2. The phosphorylation of ERK and JNK was observed when *U. urealyticum* was added to PMCs and knock out of Tlr2 inhibited these MAPKs phosphorylation. Based on our co-culture study, *U. urealyticum*-infected PMCs exhibited significantly increased attachment to ESCs compared with uninfected PMCs. Collectively, *U. urealyticum* infection promotes the development of endometriosis by increasing inflammatory mediators, adhesion molecules, and MMP-2 expression in PMCs through TLR2 signaling. Through our results, we present a theory that infection-induced pelvic inflammation contributes to the initiation and progression of endometriosis. Appropriate treatment of reproductive tract infection may decrease the prevalence of endometriosis.

## Introduction

Endometriosis is a chronic gynecological disorder, characterized by the presence of ectopic endometrial tissue outside the uterine cavity; it affects 6–10% of women of reproductive age (1). Further, its frequency in women with chronic pelvic pain or infertility ranges from 35 to 50% (1).

Among several hypotheses, Sampson's theory of retrograde menstruation is still applicable (2). Some other theories such as alteration of response to estrogen and progesterone, endometrial stem cell implantation, Müllerian remnant abnormalities, and coelomic metaplasia have postulated (3, 4). Recent studies have also reported the importance of interactions among endometrial tissue, the peritoneum, and immune cells during development into endometriosis (5–7). In the early development stage of endometriosis, acute inflammatory responses and tissue breakdown were induced by pro-inflammatory cytokines such as interleukin (IL)-6, IL-1β, interferon (IFN)-γ, and tumor necrosis factor (TNF). As inflammation progresses, tissue remodeling and repair, endometrial cell proliferation, angiogenesis, neurogenesis, and fibrosis are followed under the influence of regulatory T cells and T helper 2 cells (7–10).

According to recent studies, the menstrual blood of women with endometriosis is more frequently contaminated with *Escherichia coli* than that of controls, which corresponded to higher levels of endotoxin in the menstrual fluid (11, 12). In addition, lipopolysaccharide (LPS) promotes the proliferation and invasion of human endometrial stromal cells via the upregulation of cyclooxygenase-2 (COX-2) and prostaglandin E2 (PGE2), which can result in development into endometriosis (13). Moreover, based on an epidemiological study performed in Taiwan, endometriosis is more prevalent in women with low genital tract infection of the cervix, vagina, and vulva compared to that in women without disease (14).

*Ureaplasma urealyticum* is a gram-negative bacterium belonging to the family Mycoplasmataceae that has no cell wall. This species is an important opportunistic pathogen commonly found in the reproductive organs of sexually active females, and its prevalence ranges from 60 to 80% worldwide (15–17). *U. urealyticum* is involved in a variety of infectious diseases such as non-gonococcal urethritis, male infertility, bacterial vaginosis, chronic endometritis, pelvic inflammatory disease, spontaneous abortion, premature birth, and chorioamnionitis (18–21). However, the role of this bacterium in the progression of endometriosis has not been described.

The peritoneum, a common ectopic endometrial implantation site, is composed of a wide monolayer of mesothelial cells. Peritoneal mesothelial cells (PMCs) cover the body's serous cavity and internal organs (22). These PMCs participate in diverse cellular processes including tumor cell adhesion, tissue repair, inflammation, and host defense (22, 23). The sensing of bacterial pathogens in PMCs is mediated by some Toll-like receptors (TLRs) including TLR2 (23) which recognizes a molecular pattern of *U. urealyticum* (15, 24, 25). Further, in humans and mice, stimulated PMCs secrete several CC and CXC chemokines and cytokines including CXCL1/KC, CCL2/MCP-1, and IL-6 (26–29).

Based on this previous knowledge, we hypothesized that *U. urealyticum* infection might contribute to the development of endometriosis by inducing the production of inflammatory mediators by PMCs, possibly through TLR2. Hence, our objective was to reveal whether PMC infection by *U. urealyticum* is associated with endometriosis. Moreover, we aimed to demonstrate the molecular mechanism involved in the development of endometriosis.

## Materials and Methods

### Mice

Wild-type (WT) C57BL/6J female mice were purchased from DBL (Eumseong, South Korea). Tlr2-deficient female mice in a C57BL/6 background were purchased from Jackson Laboratories (Bar Harbor, ME, USA). The animals were housed in an animal room at a constant temperature (22–24°C) and light–dark cycle with 14 h of light and 10 h of dark. Food and water were available *ad libitum*. Mice were acclimatized to the laboratory room for 1 week before the experiment. Mice were sacrificed by cervical dislocation; female C57BL/6J (*n* = 92) and Tlr2-deficient mice (*n* = 31) of 4–8 weeks of age were used for this study. Animal studies were approved and conducted according to the regulations of the Institutional Animal Care and Use Committee (IACUC; approved protocol number: P-17-09-E-01) at Konyang University (Daejeon, Korea).

### *U. urealyticum* Culture

*Ureaplasma urealyticum* (ATCC 27618) was reconstituted in American Type Culture Collection media 2616, in accordance with the instructions provided by the American Type Culture Collection. Bacteria were incubated under anaerobic conditions at 37°C until the medium changed from yellow to pink–red. The color change indicates *U. urealyticum* growth. After 12 h of color change, bacteria were gently pelleted and resuspended in growth medium. The bacterial concentrations of the suspensions were adjusted to 1 × 10^4^ CFU/mL using the Mycoplasma IST-2 kit (BioMerieux, Marcy l'Etoile, France), according to the manufacturer's instructions (30). Next, we identified colonies using A7 Mycoplasma Agar (BioMerieux) to determine the exact number of bacteria. *U. urealyticum* was incubated on A7 Mycoplasma Agar for 24 h under anaerobic conditions using gaspak EZ large incubation (BD Biosciences, San Jose, CA, USA). The growth of *U. urealyticum was* confirmed microscopically. Colonies of *U. urealyticum* appeared granular and dark brown. At the end of culture, *U. urealyticum*-positive plates changed color from orange to red, and the presence of *U. urealyticum* colonies was morphologically confirmed under a microscope.

### Mouse Model of Endometriosis

**Figure 1:**
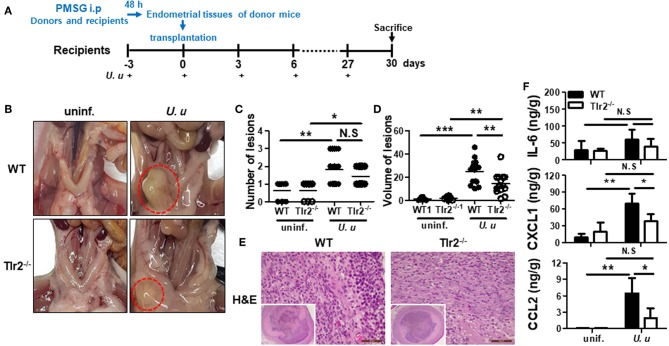
Experimental design and characterization of ectopic lesions in a mouse model of endometriosis induced by *U. urealyticum* infection. The experimental design of the endometriosis mouse model is shown. Sixteen uninfected mice were used in total; 8 WT and 8 Tlr2^−/−^ mice, each. Also, 32 *U. urealyticum* infected mice were used; 16 WT and 16 Tlr2^−/−^ mice, each **(A)**. Images show the ectopic lesions harvested 30 days after the induction of endometriosis **(B)**. Dotted lines indicate the ectopic lesions near the uterus **(B)**. The average numbers and volume of the ectopic lesions are shown **(C,D)**. H&E stain images (40x and 400x) are also presented **(E)**. The concentrations of IL-6, CXCL1, and CCL2 in culture supernatants of harvested ectopic endometriotic lesions were measured by ELISA **(F)**. All experiments were analyzed by a *t*-test and expressed as the mean ± SD. ^*^P < 0.05; ^**^P < 0.01; ^***^P < 0.001. ^−/−^, knockout; N.S, not significant; uninf., uninfected; *U. u, U. urealyticum*.

We developed an animal model of endometriosis by modifying the method of Hirata et al. (31). Briefly, both 6- to 8-week-old donor (WT mice, *n* = 48) and recipient female mice (WT, *n* = 24 and Tlr2-deficient mice, *n* = 24) were primed with pregnant mare serum gonadotropin (PMSG) (Prospec, East Brunswick, NJ, USA) at 5 IU/mouse for 48 h to stimulate endometrial growth and synchronize the estrus cycle. After 48 h, the donor mouse was sacrificed by cervical dislocation and the uterine horns were removed and put in a dish containing cold PBS. After peeling off the serosa and myometrium gently, endometrial tissue was minced with a razor blade. The endometrial fragments were suspended in 0.5 mL PBS and injected into the peritoneal cavities of recipient mice with an 18-gauge needle at a 1:1 ratio (donor to recipient). Three days before the induction of endometriosis, the recipients (16 uninfected mice: 8 WT and 8 Tlr2^−/−^ mice, each and 32 *U. urealyticum* infected mice: 16 WT and 16 Tlr2^−/−^ mice, each) were injected with bacteria (1 × 10^6^/200 μL/mouse) into the peritoneal cavities. This injection was repeated every 3 days until the 27th day after endometrial cell injection, 11 times in total (Figure 1A). The recipient mice were euthanized 30 days after the induction of endometriosis. The number, location, and sizes of ectopic lesions were assessed under a dissecting microscope, which was followed by histological evaluation. Sizes of ectopic lesions were measured based on two perpendicular diameters using calipers. The lesion volume was calculated using the formula V = (4/3)πr_1_^2^ r_2_ (r_1_ and r_2_ are radii, r_1_ < r_2_, as described previously) (32).

### Histological Staining

Formalin-fixed specimens (uterine tissues and ectopic lesions) obtained from the modeled mice were paraffin-embedded, cut into 4-μm sections, and stained with hematoxylin and eosin stain (H&E stain). Sections were examined for the presence of histologic hallmarks of endometriosis under a microscope.

### Preparation and Stimulation of Mouse Peritoneal Mesothelial Cells

PMCs were isolated mainly from the peritoneum and some from external surfaces of the liver, spleen, and kidneys of 4-week-old WT (*n* = 12) and Tlr2-deficient (*n* = 7) female mice as described previously (27). This method can reduce the sacrificed number of PMC donor mice. Briefly, pieces of tissue were obtained from sacrificed mice and washed by injecting ~50 mL of phosphate-buffered saline (PBS; pH 7.4) and digested with 0.25% trypsin/EDTA solution (Gibco, Basel, Switzerland) for 50 min at 37°C. After discarding intact tissues and tissue debris, the cell suspension was centrifuged at 500 × *g* for 5 min. The pellet was resuspended in Dulbecco's modified Eagle's medium (DMEM; Invitrogen, Grand Island, NY, USA) supplemented with 15% FBS (Invitrogen), 2 mM L-glutamine (Gibco), and 1% penicillin–streptomycin (Gibco) and cultured overnight. The next day, floating cells were removed and adherent cells were cultured for 3 additional days. PMCs were seeded in 48-well plates at a concentration of 2 × 10^5^ cells/well or in 6-well plates at a concentration of 1 × 10^6^ cells/well and incubated in a 5% CO_2_ incubator at 37°C. The day after seeding, cells were treated with Pam_3_CSK_4_ (InvivoGen, San Diego, CA, USA), LPS (InvivoGen), or *U. urealyticum* at the indicated multiplicity of infection (MOI).

### Enzyme-Linked Immunosorbent Assay

The concentrations of IL-6, CXCL1, and CCL2 in culture supernatants of PMCs, endometrial stromal cells (ESCs), and ectopic lesions were determined using a commercial enzyme-linked immunosorbent assay (ELISA) kit (R&D Systems, Minneapolis, MN, USA) and a 450 nm plate reader (Bio Tek, Winooski, VT, USA).

### Western Blotting

PMCs were lysed in ice-cold RIPA buffer (Elpis Biotech, Daejeon, Korea) containing a protease inhibitor cocktail (Thermo Fisher Scientific, Waltham, Massachusetts, USA) and phosphatase inhibitor (Thermo Fisher Scientific). For cell lysates, standard western blotting was performed (33) as described using antibodies against MMP-2 and total and phospho-ERK, JNK, and p-38 (Cell Signaling Technology, Beverly, MA, USA). Immunofluorescent-labeled antibody to rabbit IgG (Cell Signaling Technology) was used to visualize western blots with the Fusion solo S chemidoc (Vilber, Collégien, France).

### Flow Cytometry

PMCs were seeded in 6-well plates at a concentration of 1 × 106 cells/well and were infected with *U. urealyticum* (MOI 0.1) for 6, 12, and 24 h. Cells were stained with the following monoclonal antibodies (mAbs; BioLegend Inc., San Diego, CA, USA): fluorescein isothiocyanate (FITC)-conjugated anti- intercellular adhesion molecules-1 (ICAM-1; CD54) and anti- vascular cell adhesion molecule-1 (VCAM-1; CD106) mAbs (0.25 μg/10^6^ cells in 100 μL, for each). Isotype-matched controls were run in parallel. A total of 10,000 cells were counted. Viable lymphocytes were gated based on their forward- and side-scatter profile. The samples were acquired using a BD FACSCalibur Flow Cytometer (BD Biosciences) and the data were analyzed using BD CellQuest Pro software (BD Biosciences).

### Attachment Assay and Confocal Microscopy

PMCs labeled with CellTracker Orange (Molecular Probes, Eugene, OR, USA) at 10 μM for 30 min were seeded in 35-mm confocal dishes (SPL, hole size; 20 mm) at a concentration of 1.5 × 10^6^ cells/well, and incubated in a 5% CO_2_ incubator at 37°C. The day after seeding, cells were stimulated with *U. urealyticum* at an MOI of 0.1 for 12 h. PMCs were washed twice with PBS to remove uninfected bacteria. For the co-culture system, ESCs were stained with CellTracker Green (Molecular Probes) at 10 μM for 30 min and seeded in dishes with prepared PMCs at a concentration of 1 × 10^5^ cells/well. These cells were then incubated for three different amounts of time, specifically 15, 30, and 60 min. The cells were fixed with 4% paraformaldehyde (Sigma-Aldrich, St. Louis, MO, USA) for 10 min and then analyzed using a confocal microscope, LSM 700 (Zeiss, Jena, Germany) and ZEN 2012 black edition software.

In addition, we performed attachment assays with an immortalized human mesothelial cell line, MeT-5A (a kind gift from Dr. D.Y. Kim at Seoul National University, Seoul, Korea), and an immortalized human endometrial stromal cell line, T-HESCs (ATCC CRL-4003). These cells were stained with CellTracker Orange (MeT-5A) and CellTracker Green (T-HESC), respectively. We treated MeT-5A cells with Pam_3_CSK_4_ (1 μg/mL) instead of *U. urealyticum*. The cells were processed as described previously herein.

### Isolation and Culture of Mouse Endometrial Stromal Cells

Endometrial cells were isolated from the uterus of WT female mice at 5–6 weeks of age. First, uteri were removed and cut lengthwise (34). The uteri from 8 mice were pooled and incubated with 0.25% trypsin (Gibco) and 2.5% pancreatin (Sigma-Aldrich) for 60 min at 4°C and 60 min at 22°C. Following transfer to 15 mL ice-cold (4°C) Hank's Balanced Salt Solution (HBSS; Gibco), digested uteri were vortexed to release epithelial cells. Uterine tissues were rinsed and vortexed additionally three times, and the resulting cell suspensions were pooled. After the removal of epithelial cells, the pooled uteri were incubated for 30 min at 37°C in HBSS with 0.02% EDTA, 0.05% trypsin, and 400 U/mL DNase (Gibco). After adding 2 mL FBS, the cell suspension was filtered through a 40-micron nylon mesh (Falcon) to remove debris and centrifuged at 500 × *g* for 10 min. The cells were resuspended in DMEM (without phenol red)/Hams F-12 nutrient mixed 1:1, supplemented with 20 mM Hepes, 100 mg/mL streptomycin, 100 U/mL penicillin, 2 mM L-glutamine, and 10% FBS (all from Gibco). After 4 days of culture, non-adherent cells were removed by changing the medium at 48 h intervals, and these adherent ESCs were used for ELISA and co-culture studies.

### Statistical Analysis

The results of ELISA, western blotting, and flow cytometry were analyzed using repeated measures ANOVA, followed by multiple comparisons using Tukey's procedure. The average numbers and volume of the ectopic lesions were compared between two groups using *t*-test. Statistical analysis of the data was performed using GraphPad Prism version 5.00 (GraphPad Software, Inc., La Jolla, CA, USA). Data are presented as mean ± standard deviation (SD). Values of *P* < 0.05 or less were considered statistically significant.

## Results

### *U. urealyticum* Infection Enhances the Development of Ectopic Lesions in a Mouse Model

To verify the hypothesis that *U. urealyticum* infection in the peritoneal cavity is involved in the development of endometriosis, PMSG was injected into the peritoneal cavity of donor and recipient mice synchronized in their estrous cycles. *U. urealyticum* was injected into the peritoneal cavity of recipient mice at 3-day intervals from 3 days before endometriosis induction (Figure 1A). When inspecting the peritoneal cavity, there was no evidence of severe acute inflammation such as adhesions of intraperitoneal organs and abscess formation. Endometriosis-like lesions developed near the uterus (Figure 1B). Further, more ectopic lesions developed in the group infected with *U. urealyticum* than in the uninfected group (*P* < 0.01), but there was no difference between infected WT and Tlr2-deficient mice (Figure 1C). The volume of these ectopic lesions was significantly larger in the group infected with *U. urealyticum* than in the uninfected groups comprising WT and Tlr2-deficient mice (*P* < 0.001 and 0.01, respectively; Figure 1D). In particular, the volumes of ectopic lesions were larger in WT mice than in Tlr2-deficient mice (*P* < 0.01; Figure 1D).

Next, to further characterize the ectopic lesions obtained from our mouse model of endometriosis, lesions were subjected to histological examination by H&E stain. As shown in Figure 1E, these lesions were filled with inflammatory cells and luminal epithelial cell debris.

Thus, we investigated the inflammatory response in ectopic lesions caused by *U. urealyticum* infection. As a result, infected WT mice produced higher levels of CXCL1 and CCL2 than uninfected WT (*P* < 0.01, for each) and infected Tlr2-deficient (*P* < 0.05, for each) mice (Figure 1F). However, there was no significant difference in IL-6 production of infected WT mice compared to those of uninfected WT and infected Tlr2-deficient mice. From this endometriosis model, we also found that the intraperitoneal infection of *U. urealyticum* results in the development of ectopic lesions.

### *U. urealyticum* Induces the Production of IL-6, CXCL1, and CCL2 in PMCs and Endometrial Stromal Cells

PMCs were stimulated with *U. urealyticum* at MOIs of 0.01, 0.05, and 0.1 for three different durations (6, 12, and 24 h) and a cytokine and chemokines were measured by ELISA. *U. urealyticum* infection led to the dose-dependent production of IL-6, CXCL1, and CCL2 in PMCs at every time point (*P* < 0.01 or < 0.001, at an MOI 0.1) (Supplementary Figure 1). Similarly, when ESCs were stimulated with different MOIs (0.01–0.1) of *U. urealyticum* for 24 h, the productions of IL-6, CXCL1, and CCL2 were significantly increased in a dose-dependent manner as compared with uninfected ESCs (Supplementary Figure 2). This indicates that *U. urealyticum* can induce an inflammatory response by PMCs and ESCs.

### *U. urealyticum* Increases the Secretion of IL-6, CXCL1, and CCL2 in PMCs via the TLR2-Dependent Pathway

Previous reports showed that *U. urealyticum* possesses molecular patterns that can be recognized by TLR2. We thus investigated whether *U. urealyticum* could promote cytokine and chemokine production by PMCs via TLR2 activation.

**Figure 2:**
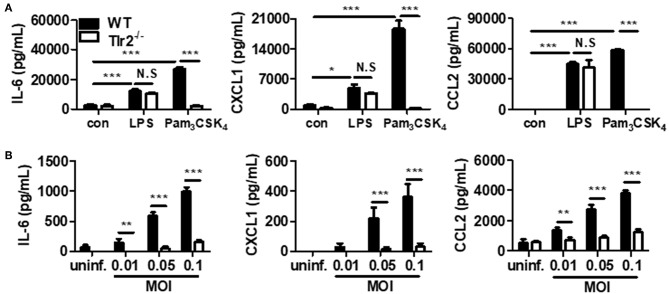
*U. urealyticum* increases the production of cytokines and chemokines in PMCs via TLR2. WT and Tlr2-deficient PMCs were incubated with Pam_3_CSK_4_ (100 ng/mL) and LPS (10 ng/mL) for 24 h **(A)**. Different MOIs of *U. urealyticum* were applied and PMCs were incubated for 6 h **(B)**. The concentrations of IL-6, CXCL1, and CCL2 in the culture supernatant were measured by ELISA. Data are shown as the mean ± SD of triplicate samples from one experiment representative of three independent experiments (^*^P < 0.05; ^**^P < 0.01; ^***^P < 0.001). ^−/−^, knockout; con, control; uninf., uninfected; N.S, not significant.

First, we compared the characteristics of PMCs obtained from WT and Tlr2-deficient mice. WT and Tlr2-deficient PMCs were treated with two types of TLR ligands (TLR2 ligand, Pam_3_CSK_4_; TLR4 ligand, LPS). Regarding the IL-6, CXCL1, and CCL2 production, Pam_3_CSK_4_ increased the secretion of all three molecules from WT PMCs, but not from Tlr-2 deficient PMCs (*P* < 0.001, for each). In contrast, there was no significant difference in the production of IL-6, CXCL1, and CCL2 after LPS stimulation between WT and Tlr2-deficient PMCs (Figure 2A). These results suggested that Tlr2-deficient PMCs are deficient in TLR2 signaling and WT PMCs produce significant levels of inflammatory mediators upon stimulation by Pam_3_CSK_4_.

Next, we compared the production of a cytokine and chemokines between WT and Tlr2-deficient PMCs upon *U. urealyticum* infection. After 6 h of infection, the production of IL-6, CXCL1, and CCL2 increased in WT PMCs, but not in Tlr2-deficient PMCs (*P* < 0.001, for each at MOIs of 0.05 and 0.1; Figure 2B). Further, upon *U. urealyticum* infection at an MOI 0.01, the production of IL-6 and CCL2, but not CXCL1, was elevated (*P* < 0.01, each). These observations indicated that *U. urealyticum* infection induces a pro-inflammatory response in PMCs via the TLR2-dependent pathway.

### *U. urealyticum* Activates ERK and JNK Pathways in PMCs

**Figure 3:**
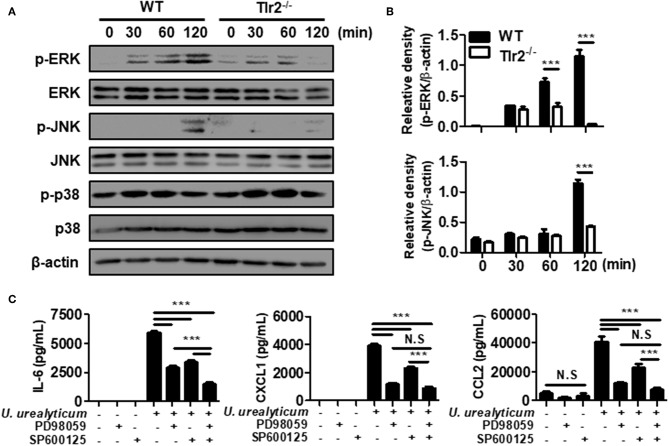
*U. urealyticum* enhances the activation of MAPKs in PMCs. WT and Tlr2-deficient PMCs were infected with *U. urealyticum* at an MOI of 0.1, and protein was extracted at the indicated time points. The phosphorylation of ERK, JNK, and p-38 was analyzed by western blotting **(A)**. The relative density of ERK and JNK phosphorylation following *U. urealyticum* infection was compared to that of β-actin **(B)**. After pre-treating WT PMCs with 40 μM of an ERK inhibitor (PD98059) and 20 μM of a JNK inhibitor (SP600125) for 2 h, the cells were infected with *U. urealyticum* at an MOI of 0.1 for 6 h **(C)**. The concentrations of IL-6, CXCL1, and CCL2 in culture supernatants were measured by ELISA. Data are shown as the mean ± SD of triplicate samples from one experiment representative of three independent experiments (^***^P < 0.001). ^−/−^, knockout; N.S, not significant.

In response to bacterial infection, TLRs of the innate immune system release inflammatory mediators via the mitogen-activated protein kinase (MAPK) signaling pathways (35). To investigate whether MAPK signaling is involved, we infected WT and Tlr2-deficient PMCs with *U. urealyticum* at an MOI of 0.1. In WT PMCs, ERK was phosphorylated at 30, 60, and 120 min, whereas JNK was phosphorylated at 120 min post-infection. However, ERK and JNK phosphorylation was significantly abolished in Tlr2-deficient PMCs (Figures 3A,B). There were no significant differences in p38 phosphorylation between WT and Tlr2-deficient PMCs in response to *U. urealyticum* infection (Figure 3A). Next, we performed an inhibitor assay to determine whether ERK and JNK pathways are important to induce pro-inflammatory conditions in PMCs exposed to *U. urealyticum*. Inhibitors of ERK (PD98059) and JNK (SP600125) suppressed the production of IL-6, CXCL1, and CCL2 in *U. urealyticum*-infected WT PMCs (Figure 3C). These results indicated that *U. urealyticum* enhances the production of IL-6, CXCL1, and CCL2 via the ERK and JNK pathways in PMCs.

### *U. urealyticum* Enhances the Expression of ICAM-1 and VCAM-1

**Figure 4:**
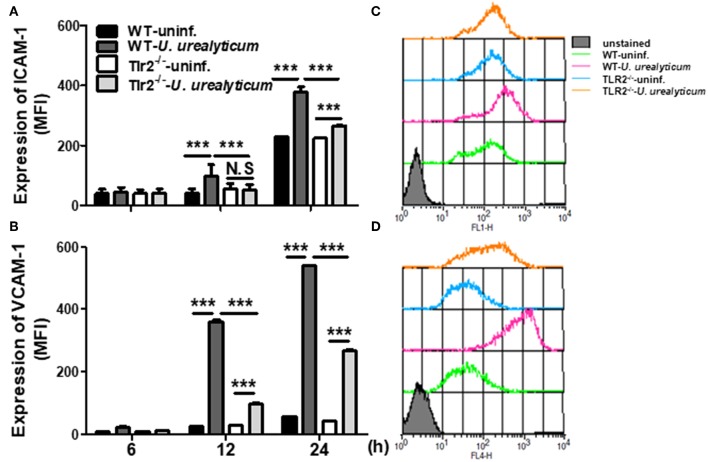
*U. urealyticum* infection increases the expression of adhesion molecules in PMCs. WT and Tlr2-deficient PMCs were infected with *U. urealyticum* (MOI of 0.1) for 6–24 h. The expression of ICAM-1 **(A)** and VCAM-1 **(B)** was analyzed by flow cytometry. Representative plots of ICAM-1 **(C)** and VCAM-1 **(D)** expression are shown. All experiments were performed in triplicate. Data are shown as the mean ± SD (*n* = 3). ^***^P < 0.001 compared to respective groups. N.S, not significant; ^−/−^, knockout; uninf., uninfected; MFI, mean channel fluorescence intensity.

The expression of cell adhesion molecules such as ICAM-1 and VCAM-1 plays a vital role in the development of endometriosis, especially in the early stages of pathogenesis (36, 37). Accordingly, we investigated whether *U. urealyticum* considerably affects the expression of these markers in PMCs. Compared with the uninfected WT PMCs group, WT PMCs increased the expression of ICAM-1 and VCAM-1 by *U. urealyticum* infection at 12 and 24 h (*P* < 0.001, for each; Figures 4A,B). In infected Tlr2-deficient PMCs, those molecules were also upregulated at 24 h compared to levels in uninfected Tlr2-deficient PMCs group (*P* < 0.001, for each; Figures 4A,B), but these increases were diminished compared to those observed for infected WT PMCs group (*P* < 0.001, for each; Figures 4A,B). These results implied that *U. urealyticum* significantly upregulates ICAM-1 and VCAM-1 expression in PMCs, and that several mechanisms including the TLR2 pathway might control the expression of these adhesion molecules.

### *U. urealyticum* Enhances the Expression of MMP-2 in PMCs

**Figure 5:**
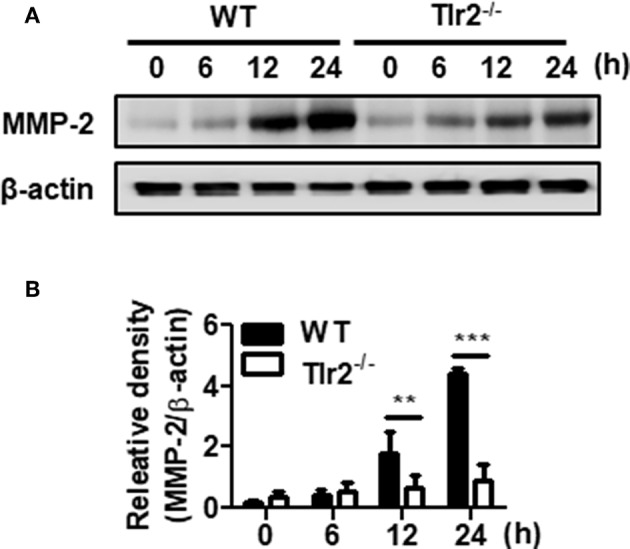
*U. urealyticum* enhances the expression of MMP-2 in PMCs. WT and Tlr2-deficient PMCs were infected with *U. urealyticum* (MOI of 0.1). Protein was extracted at the indicated time points. The expression of MMP-2 was examined by western blotting **(A)**. The relative density of MMP-2 activation following *U. urealyticum* infection was compared to that of β-actin **(B)**. Data are shown as the mean ± SD (*n* = 3). ^**^P < 0.01; ^***^P < 0.001. ^−/−^, knockout.

Matrix metalloproteinases (MMPs) play a crucial role in tissue remodeling associated with various pathological or physiological processes such as the infiltration and dissemination of malignant tumors or placental trophoblasts (38). We next determined whether *U. urealyticum* modulates MMP expression in PMCs. As shown in Figure 5, *U. urealyticum* infection significantly increased the expression levels of MMP-2 in WT PMCs at 12 (*P* < 0.01) and 24 h (*P* < 0.001) compared to those in Tlr2-deficient PMCs. However, expression of MMP-2 was not enhanced by *U. urealyticum* infection in Tlr2-deficient PMCs. Our results show that *U. urealyticum* enhances the expression of active MMP-2 in PMCs and that this is blocked in Tlr2-deficient PMCs.

### The Attachment of Endometrial Stromal Cells to Mesothelial Cells Is Facilitated by *U. urealyticum* Infection Based on a Co-culture System

We further investigated whether *U. urealyticum* modulates ESCs adhesion to PMCs using our co-culture system. For this, we infected mesothelial cells with *U. urealyticum* for 12 h to induce expression adhesion molecules and MMP and then co-incubated endometrial stromal cells with infected mesothelial cells.

**Figure 6:**
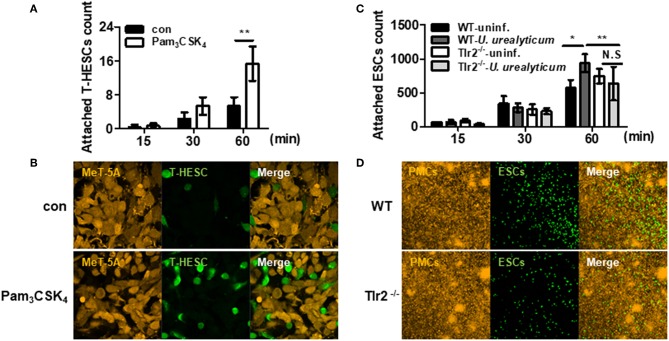
*U. urealyticum* promotes the attachment of endometrial stromal cells to mesothelial cells via TLR2. The MeT-5A and PMCs were stimulated with or without Pam_3_CSK_4_ and *U. urealyticum*. After a 12 h infection, T-HESC and ESCs were incubated for 15, 30, and 60 min **(A–D)**. The attached T-HESC and ESCs were enumerated using ImageJ software **(A,C)**. Images were obtained after 60 min with a 100x **(B)** and 10x **(D)** objective by confocal laser scanning microscopy. All experiments were performed in triplicate. Data are shown as the mean ± SD (*n* = 3). ^*^P < 0.05; ^**^P < 0.01. N.S, not significant; ^−/−^, knockout. uninf., uninfected.

In an experiment with human mesothelial cells (MeT-5A) and human endometrial stromal cells (T-HESC), TLR2 ligand, Pam_3_CSK_4_, increased the attachment of T-HESC to MeT-5A compared to that in the untreated group (*P* < 0.01; Figures 6A,B). We further performed an attachment experiment with ESCs and PMCs obtained from mice. ESC attachment was significantly increased upon co-culture with infected WT PMCs compared to that with uninfected WT PMCs (*P* < 0.05; Figure 6C). Moreover, this attachment was increased in infected WT cells compared to that with infected Tlr2-deficient PMCs (*P* < 0.01; Figures 6C,D). However, there was no significant difference for Tlr2-deficient PMCs with or without *U. urealyticum* infection (Figure 6C). Our results indicated that *U. urealyticum* facilitates the attachment of ESCs to PMCs via TLR2 signaling.

## Discussion

In this study, we investigated whether pelvic infection can cause endometriosis and which mechanism is involved. Many previous reports regarding the pathogenesis of endometriosis have demonstrated abnormal features of the endometrium itself, hormone biosynthesis, immune cells, angiogenesis, neurogenesis, and cell death (4, 5, 39). However, the role of PMCs has been underestimated (40). Furthermore, recently, peritoneal inflammation triggered by bacteria arose as a new possible pathogenesis of endometriosis. Several studies have suggested a connection between bacterial infection in the female genital tract and endometriosis (11, 14, 41, 42). Therefore, new insights into the role of the peritoneal microenvironment with bacterial infections will provide a better understanding of endometriosis. *U. urealyticum* is one of the most common commensal microorganisms residing in the female reproductive tract and causes many obstetric and gynecological disorders (15–17). However, the importance of *U. urealyticum* infection in terms of endometriosis has been overlooked to date. Thus, we hypothesized that peritoneal inflammation and the breakdown of its integrity caused by *U. urealyticum* might contribute to the initiation and progression of pelvic endometriosis, probably through TLR2.

Based on our newly-developed *in vivo* mouse model, the number and size of ectopic lesions was significantly increased in *U. urealyticum*-infected mice compared to those in control mice (Figure 1). Our *in vitro* studies represented the PMCs response to *U. urealyticum* infection and the interaction between ESCs and PMCs at the initial stage of endometriosis development. Meanwhile, the *in vivo* animal study models a subsequent stage of endometriosis progression since ectopic lesions were observed 30 days post transplantation. Recently, Azuma et al. (12) reported that the repeated intraperitoneal injection of bacterial endotoxin (LPS) could induce chronic peritoneal inflammation and reproduce endometriosis in mice. However, that study induced endometriosis with endotoxin, but not with live bacteria. To our best knowledge, this is the first animal model of endometriosis triggered by intraperitoneal microbial infection.

Here, in assessing the interaction between *U. urealyticum* and PMCs, we showed that this bacterium could increase not only the production of IL-6, CXCL1, and CCL2, but also the expression of ICAM-1, VCAM-1, and MMP-2 from PMCs. There is a body of evidence that shows the relationship between PMC inflammation and development of endometriosis. *In vitro* experiments demonstrated endometrial fragment attached to only the site of damaged peritoneum with exposure of basement membrane or extracellular matrix (43). Moreover, inflammatory mediators (cytokines, chemokines, and growth factors) are aberrantly expressed in the peritoneal fluid of women with endometriosis (44–48). This local inflammatory microenvironment promotes the growth and maintenance of ectopic lesions through endometrial–peritoneal adhesion, invasion, angiogenesis, and proliferation via loss of tight junction, MMP, and epithelial-mesenchymal transition (40, 49).

The concentrations of IL-1β, IL-1 converting enzyme, IL-8, and regulated on activation, normal T-cell expressed and secreted (RANTES) were increased in the peritoneal fluid of patients with endometriosis (45, 47). Similarly, IL-1β, IL-6, TNF-α, and prostaglandin E_2_ (PGE2) were found to be rich in the peritoneal fluid of women with endometriosis (44, 46).

During the development of endometriosis, these cytokines and chemokines play a role. IL-6 is a pro-inflammatory cytokine that activates T and B cells and facilitates the recruitment of macrophages (5, 40). IL-8, a chemoattractant for macrophages and neutrophils, is vigorously secreted from the peritoneum of women with endometriosis upon stimulation by IL-1 and TNF-α (50). Since rodents do not have an IL-8 homolog, it is assumed that mice compensate for the absence of IL-8 with the ligands CXCL1 and CXCL2 (51). CXCL1 has neutrophil chemoattractant activity via CXCR2 binding (52), but has been scarcely studied with respect to endometriosis. CCL2 recruits macrophages and might be involved in the growth and maintenance of ectopic endometrium to secrete cytokines and growth factors and directly stimulate the proliferation of endometrial cells (53). Levels of CCL2 were previously found to be significantly correlated with the stage of endometriosis (54) and were higher in women with endometriosis than in women with tubal infertility (55). Similarly, in the peritoneal fluid of women with endometriosis, levels of CXCL1, CXCL2, CCL2, MCP-3, and HGF were found to be significantly elevated compared to those in control individuals (40, 53, 56).

However, most clinical studies have measured cytokines from peritoneal fluid, which could be influenced by various components such as PMCs, immune cells, and ectopic lesions (40). In this study, we observed the increased production and secretion of IL-6, CCL2, and CXCL1 from PMCs in response to *U. urealyticum* infection. These results are in line with suggestion from others that PMCs might actively participate in the generation of a pro-inflammatory environment in the peritoneum (40, 49). For the development of endometriosis, the adhesion and attachment of endometrial cells to the pelvic mesothelium is a critical step (57). Some adhesion molecules such as integrins α2β1 and α3β1, P-cadherin, ICAM-1, and VCAM-1 have been suggested to be key factors regulating the attachment of endometrial cells (40, 58, 59). Even though pro-inflammatory cytokines are known to upregulate these adhesion molecules (40), it is not clear whether those molecules increase when PMCs are infected by *U. urealyticum*. We observed the significant upregulation of ICAM-1 and VCAM-1 in PMCs following *U. urealyticum* infection. Their increased expression could provide suitable sites for the attachment of endometrial cells.

Next, we investigated MMP production from PMCs since the invasion of endometrial cells into the pelvic mesothelium is considered an essential factor for the development of endometriosis (40). Several studies have reported higher expression of MMP-2, MMP-9, MMP-14, and MMP-24 in the eutopic endometrium or ectopic lesions of women with endometriosis, as compared to levels in women without endometriosis (60–62). Peritoneal fluids and sera from endometriosis patients were also shown to contain higher levels of MMP-2 than those from controls (63). In our study, MMP-2 expression was also significantly increased in the PMCs by *U. urealyticum* infection. This peritoneal MMP expression might facilitate extracellular matrix remodeling during endometriosis development (40).

Through the co-culture experiments, we found that ESCs attached to PMCs and that *U. urealyticum-*infected PMCs showed significantly enhanced attachment to ESCs compared to uninfected PMCs. A similar co-culture examination without infection was reported by Nair et al. (64). They observed that ESCs attached to PMCs in 1 h and that ESC invasion occurred at 6 h. Through interactions between PMCs and ESCs in this co-culture system, several endometriosis-related genes including CD44, ERK, CSF-1, and c-Met were upregulated in both PMCs and ESCs. Another co-culture study with human ESCs and human PMCs showed the relaxation of PMC integrity at the beginning of ESC invasion and the loss of PMC adhesion to the substrate (65). These studies indicate that functional and morphological changes of PMCs potentiate the development of endometriosis, which might be triggered by *U. urealyticum* (15–17).

As the next step, we explored which mechanism is involved in the activation of PMCs following *U. urealyticum* infection. *U. urealyticum*-derived lipid-associated membrane proteins (LAMPs), which are lipoproteins, are considered a major factor for TLR2 activity (66). In this study, we found that *U. urealyticum* induces a series of inflammatory responses in PMCs mainly through TLR2. Moreover, Tlr2-deficient mice secreted lower levels of a pro-inflammatory cytokine (IL-6) and chemokines (CCL2 and CXCL1) and exhibited downregulated adhesion molecule (ICAM-1 and VCAM-1) and MMP-2 expression (Figures 2–5). Furthermore, ESC attachment to PMCs induced by *U. urealyticum* infection was significantly diminished in Tlr2-deficient PMCs (Figure 6). Recently, it was reported that *U. urealyticum* infection enhances TLR2-mediated IL-6 production from amniotic epithelial cells (15). Our results indicate that the host immune response against *U. urealyticum* infection is mainly mediated through TLR2.

We further assessed transcriptional factors activated in response to *U. urealyticum*. The phosphorylation of ERK and JNK was observed when *U. urealyticum* was applied to PMCs. However, these signaling events were significantly decreased in the PMCs of Tlr2-deficient mice. These results also suggest that TLR2 signaling is required for the activation of ERK and JNK MAP kinases in PMCs (Figures 1–3).

There are some limitations to our study. First, it was not performed with cells from patients with endometriosis. However, when we examined the attachment of T-HESC cells to MeT-5A cells using our co-culture system, a TLR2 ligand increased the attachment of these cells. This experiment with human cells strongly supports our results obtained from mice. Moreover, we did not investigate the presence of peritoneal *U. urealyticum* infection in women with pelvic endometriosis. If *U. urealyticum* could be isolated from the peritoneal washing or tissue, it could demonstrate a direct connection between these bacteria and disease. However, according to Kobayashi et al. (67), pelvic infection, initiating the adhesion and growth of ectopic endometrial tissue, is suppressed by the innate immunity of the host and following sterile inflammation, can continue and progress into endometriosis. Therefore, the identification of microorganisms in the pelvic cavity of women with developed endometriosis is less likely.

In summary, we observed that *U. urealyticum* infection increases inflammatory mediators, adhesion molecules, and MMP-2 expression in PMCs through TLR2 signaling and promotes endometriosis. We present a strong evidence that may support an infection theory in endometriosis development. To our best knowledge, this is the first study suggesting that *U. urealyticum* can provoke pelvic endometriosis in an animal model. Moreover, in this study, we developed a new animal model of endometriosis triggered by *U. urealyticum* infection. Through our results, we present a theory that infection-induced pelvic inflammation contributes to the initiation and progression of endometriosis. In conclusion, mesothelium inflammation induced by *U. urealyticum* might be one of the causes of pelvic endometriosis. Prevention of pelvic infection might decrease the prevalence of endometriosis. Further studies are warranted to elucidate this connection.

## Data Availability Statement

All datasets generated for this study are included in the manuscript/Supplementary Files.

## Ethics Statement

The animal study was reviewed and approved by the Institutional Animal Care and Use Committee (IACUC; approved protocol number: P-17-09-E-01) at Konyang University.

## Author Contributions

EN, DK, and SL designed and conceived the study. EN performed data acquisition. EN, DK, JL, J-SK, JP, and SL performed data analysis and interpretation. EN, JH, and SL drafted the manuscript. All authors participated in the critical revision of the manuscript.

### Conflict of Interest

The authors declare that the research was conducted in the absence of any commercial or financial relationships that could be construed as a potential conflict of interest.

## Abbreviations

ESC: endometrial stromal cell
MMP: matrix metalloproteinase
PMC: peritoneal mesothelial cell
WT: wild type.
