# Supplementary material for: Ureaplasma Urealyticum Infection Contributes to the Development of Pelvic Endometriosis Through Toll-Like Receptor 2
Source: Front Immunol. 2019 Oct 4;10:2373. doi: 10.3389/fimmu.2019.02373 (PMC6788432; doi:10.3389/fimmu.2019.02373)
Supplement: Supplementary file 1 [file Data_Sheet_1.docx]

Supplementary Material

## Supplementary Figures


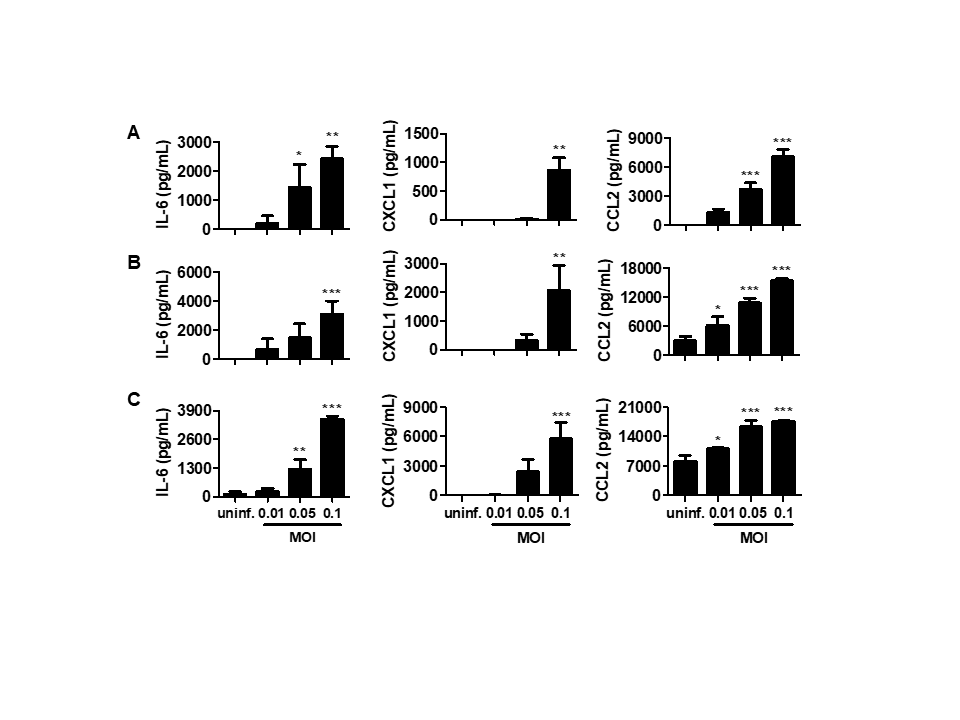


**Supplementary Figure 1.** ***U. urealyticum* induces the production of cytokines and chemokines in PMCs.** The PMCs of WT mice were stimulated with different MOIs (ranging from 0.01 to 0.1) of *U. urealyticum*, at various times including 6 (**A**), 12 (**B**), and 24 (**C**) h. The concentration of IL-6, CXCL1, and CCL2 in culture supernatants was determined by ELISA. Data are shown as the mean ± SD of triplicate samples (*P < 0.05, **P < 0.01, ***P < 0.001). MOI, multiplicity of infection; uninf., uninfected.

## Supplementary Figures


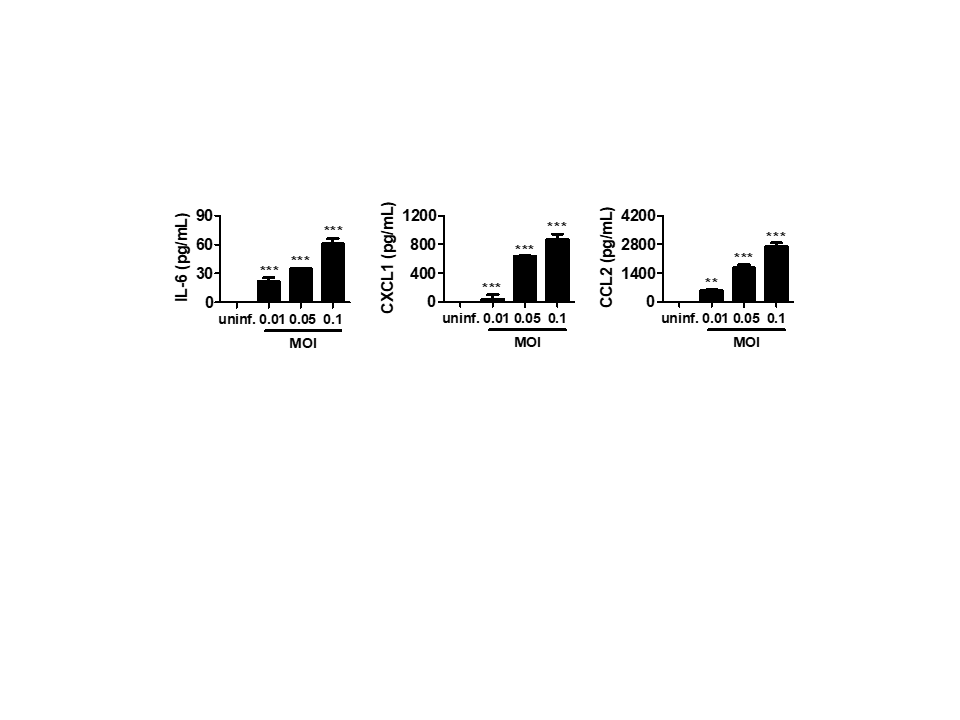


S**upplementary Figure 2. *U. urealyticum* promotes the production of cytokines and chemokines in moue primary ESCs.** The ESCs were stimulated with different MOIs (0.01 to 0.1) of *U. urealyticum*, for 24 h. The concentration of IL-6, CXCL1, and CCL2 in culture supernatants was determined by ELISA. Data are shown as the mean ± SD of triplicate samples (**P < 0.01, ***P < 0.001). MOI, multiplicity of infection; uninf., uninfected.
